# Augmented and Mixed Reality in Cardiac Surgery: A Narrative Review

**DOI:** 10.3390/jcm15031224

**Published:** 2026-02-04

**Authors:** Andreas Sarantopoulos, Maria Marinakis, Nikolaos Schizas, Dimitrios Iliopoulos

**Affiliations:** 1 Naples Comprehensive Health—Rooney Heart Institute, Naples, FL 34102, USA; 2 School of Arts and Sciences, Hunter College, New York, NY 10065, USA; maria.marinakis71@myhunter.cuny.edu; 3 Hygeia Hospital, 15123 Athens, Greece; nikschizas@gmail.com (N.S.); diliopoulos@hygeia.gr (D.I.); 4 Department of Cardiothoracic Surgery, University of Texas Health, Houston, TX 77030, USA

**Keywords:** augmented reality, mixed reality, cardiac surgery, Intraoperative navigation, robotic-assisted surgery

## Abstract

**Background:** Augmented reality (AR) and mixed reality (MR) promise to enhance anatomical understanding, spatial orientation, and workflow in cardiac surgery. Their clinical adoption remains limited and the translational path is incompletely defined. **Methods:** A PubMed search was conducted by two independent reviewers from database inception through July 2025 and identified peer-reviewed, English-language articles describing peri- or intra-operative AR/MR applications in cardiac surgery. Relevant clinical, preclinical, technical, and review articles were selected for inclusion based on scope and content. Given the narrative approach and heterogeneity across studies, findings were synthesized qualitatively into application domains. **Results:** Fourteen studies were included. Five domains emerged: (1) preoperative planning and patient-specific modelling—MR enhanced spatial orientation and planning for minimally invasive and valve procedures; (2) intraoperative navigation and visualization—AR improved targeting and interpretation with preclinical overlay errors ≈ 5 mm; (3) physiological/functional guidance—thermographic AR detected ischemia in vivo with strong correlation to invasive thermometry; (4) robotic integration and workflow optimization—AR-guided port placement and stepwise robotic adoption supported the feasibility of totally endoscopic CABG; (5) AR-based early rehabilitation. **Conclusions:** Early clinical and preclinical evidence supports AR/MR feasibility and utility for visualization and orientation in cardiac surgery. Priorities include deformable, motion-compensated registration, ergonomic integration with robotic platforms, and multicentre trials powered for operative efficiency and patient outcomes.

## 1. Introduction

Minimally invasive and robotic-assisted cardiac surgery are associated with reduced surgical trauma and recovery time at the cost of restricted visualization and diminished tactile feedback, intensifying demands on spatial orientation and decision-making [1,2]. AR/MR aims to mitigate these constraints by overlaying preoperative models or live imaging within the operative field to support planning, navigation, and communication [3,4,5]. Extended reality (XR) is used as an umbrella term encompassing augmented reality (AR), mixed reality (MR), and virtual reality (VR); throughout this review, we primarily use “AR/MR” when referring to operative applications unless a study specifically employs VR. Emerging clinical experiences suggest improvements in anatomical comprehension and workflow without prolonging procedures [5,6,7], while preclinical efforts targeting beating-heart conditions report millimetric registration and motion-compensation prototypes compatible with microscale tasks [8,9,10,11].

From a workflow perspective, contemporary minimally invasive and robotic procedures often require surgeons to integrate preoperative cross-sectional imaging (CT/MRI), intraoperative echocardiography, endoscopic video, and device telemetry across multiple displays, creating a cognitive “translation” step between 2D images and a dynamic 3D operative field. XR systems aim to reduce this gap by co-registering patient-specific models or live imaging to the surgeon’s viewpoint, thereby supporting spatial orientation, target localization, and shared situational awareness during team-based procedures [1,2,8,9].

However, the translational threshold for cardiac surgery is high: the heart is deformable and continuously moving, surgical access is constrained, and small errors may have disproportionate consequences in tasks such as leaflet repair, chordal implantation, and coronary anastomosis. As a result, the key technical questions extend beyond visualization alone to include robust deformable registration, latency and motion compensation, human-factor safety during prolonged use, and interoperability with operating-room imaging and robotic platforms [10].

Evidence in this field is expanding rapidly, with active work on multi-modal registration, motion compensation, and ergonomic display integration. This review synthesizes the current evidence base, highlighting applications, limitations, and priorities for translation.

## 2. Materials and Methods

This narrative review queried PubMed from database inception through July 2025 using a structured search combining (“augmented reality” OR “mixed reality” OR “extended reality” OR “virtual reality” OR “XR”) AND (“cardiac surgery” OR “cardiothoracic surgery” OR “cardiac surgical” OR “cardiac procedure*” OR “robotic cardiac”). Peer-reviewed, English-language articles were included if they described peri-operative or intra-operative AR/MR applications with direct and/or indirect cardiac surgical relevance. Eligible evidence included clinical feasibility or observational studies, preclinical/technical investigations with surgical relevance, and reviews directly addressing cardiac surgical applications of AR/MR. Studies with broader XR scope were included only when they addressed technical constraints or workflow considerations directly applicable to cardiac surgery (e.g., deformable registration, motion compensation, sterility/ergonomics, or integration with intraoperative imaging), and otherwise were excluded to maintain a cardiac surgical focus.

**Figure 1:**
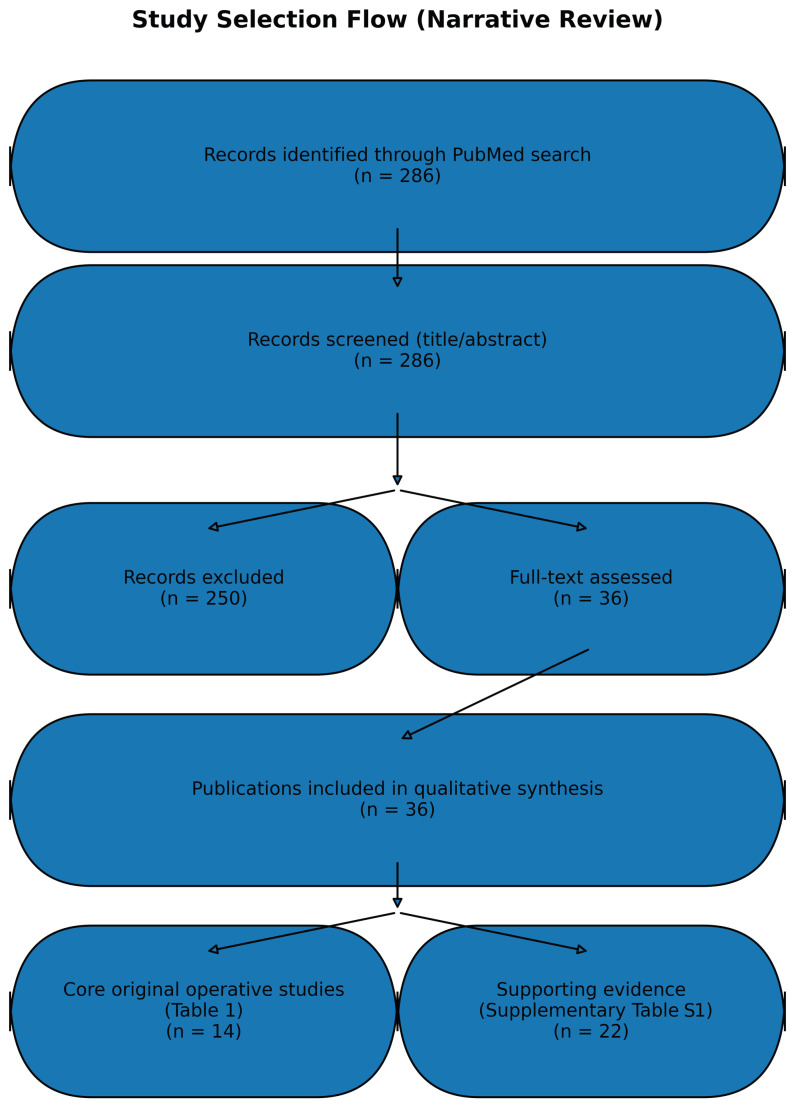
Study selection flow for the narrative review. PubMed was queried from database inception through July 2025 (*n* = 286 records). After title/abstract screening, 36 publications were included in qualitative synthesis, comprising 14 core original operative studies and 22 supporting publications.

Exclusion criteria were non-surgical cardiology applications without procedural relevance, non-cardiac operative fields without a clear cardiac surgery analogue, opinion pieces without technical or clinical content, and reports lacking sufficient description of the AR/MR system or its operative use. Titles and abstracts were screened, followed by full-text assessment of potentially eligible articles; disagreements were resolved by discussion. A simplified flow diagram summarizing identification, screening, eligibility, and inclusion has been added to improve transparency and reproducibility (Figure 1).

After that, full texts were assessed for inclusion. From each article, we extracted the study design, model or patient cohort, surgical indication, AR/MR technology and display, tracking/registration approach, accuracy metrics where available, key procedural or clinical outcomes, and stated limitations. Because this was not a systematic review and the included studies were heterogeneous in design, endpoints, and platforms, we did not perform a formal risk-of-bias assessment using standardized tools or conduct meta-analysis. Instead, we incorporated a qualitative appraisal of study design limitations, potential sources of bias, and publication bias within the Discussion to strengthen the interpretability of the evidence base. Findings were organized into thematic domains and synthesized qualitatively.

From each included publication, we extracted study type (clinical, preclinical/technical, or review), setting/model, surgical indication, XR modality (hardware and imaging source when reported), registration or visualization approach, and reported endpoints. Endpoints were categorized as objective technical or procedural measures (e.g., registration/overlay error, tool-path metrics, procedural feasibility, sizing accuracy) versus qualitative or user-reported outcomes (e.g., perceived spatial orientation, cognitive load, workflow usability). Findings were synthesized qualitatively into application domains spanning preoperative planning, intraoperative navigation/visualization, physiological guidance, workflow/robotic integration, and postoperative rehabilitation.

For the purposes of synthesis, we defined the “core” evidence set as publications with direct relevance to cardiac surgical AR/MR applications and included three evidence categories: (i) original clinical feasibility or observational studies, (ii) preclinical or technical investigations with clear operative relevance, and (iii) review articles focused specifically on AR/MR in cardiac surgery. Reviews were retained within the core set because they consolidate heterogeneous early-stage evidence, summarize platform capabilities and operative domains, and help contextualize gaps in standardization and validation. To improve transparency, we explicitly label each core publication by evidence type (clinical, preclinical/technical, or review) and distinguish primary data-generating studies from narrative syntheses in the table structure and accompanying text.

## 3. Results

This review identified 36 publications relevant to the use of augmented reality (AR) and mixed reality (MR) in cardiac surgery and perioperative care. Of these, 14 studies [1,2,3,4,5,6,7,8,9,10,11,12,13,14] constitute the core body of original preclinical and clinical investigations directly evaluating AR/MR in operative settings (Table 1), while the rest [15,16,17,18,19,20,21,22,23,24,25,26,27,28,29,30,31,32,33,34,35,36] provide supporting evidence through technical developments, patient education studies, and systematic reviews that inform cardiac surgical translation and perioperative workflow implementation (Supplementary Table S1).

**Figure 2:**
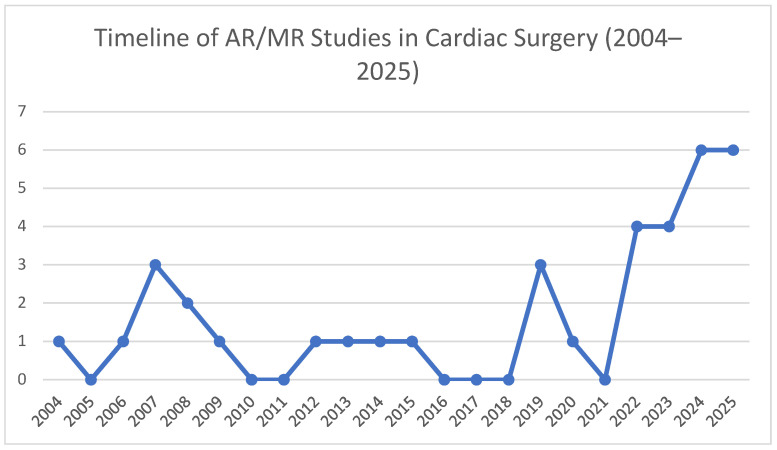
Timeline of augmented and mixed reality (AR/MR) studies in cardiac surgery (2004–2025). Line plot showing the annual number of peer-reviewed publications describing peri- or intra-operative AR/MR applications. Only sporadic feasibility studies appeared before 2010, whereas publications have increased sharply since 2019, reflecting accelerating translational activity in the field.

**Figure 3:**
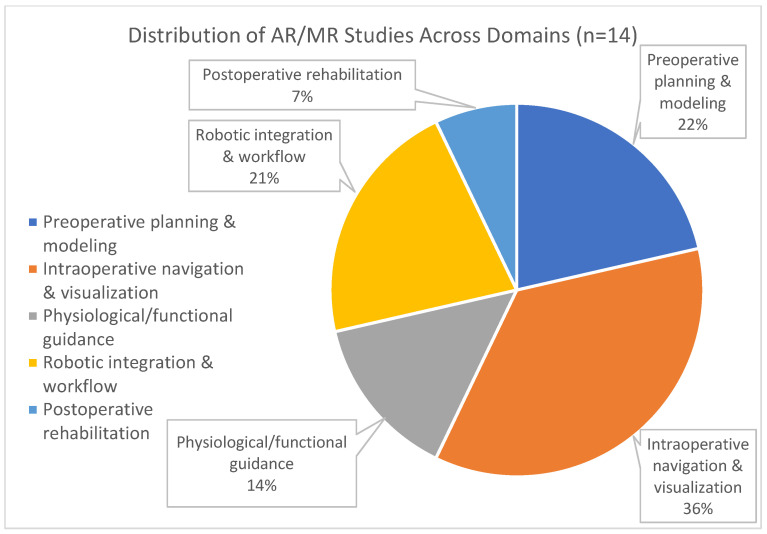
Distribution of AR/MR studies across five operative domains (*n* = 14). Pie chart illustrating the relative proportion of studies categorized as: (1) preoperative planning and modeling (3/14, 22%); (2) intraoperative navigation and visualization (5/14, 36%); (3) physiological/functional guidance (2/14, 14%); (4) robotic integration and workflow optimization (3/14, 21%); and (5) postoperative rehabilitation (1/14, 7%). Intraoperative navigation and visualization accounts for the largest share of published work.

The temporal distribution of these publications is shown in Figure 2, highlighting a marked rise in activity since 2019, while the 14 core operative studies can be further grouped into five thematic domains: preoperative planning and modeling, intraoperative navigation and visualization, physiological/functional guidance, robotic integration, and postoperative rehabilitation; their relative proportions are illustrated in Figure 3.

To provide study-level detail, core investigations reported both feasibility and quantitative technical endpoints. In the largest early clinical series, De Cannière et al. [1] scheduled 228 patients for robotic totally endoscopic coronary artery bypass grafting (117 on-pump and 111 off-pump) and completed 164 totally endoscopic cases; 28% required conversion to nonrobotic procedures, overall efficacy was 97% (angiographic patency or absence of ischemia on stress testing), and major adverse cardiac events within 6 months occurred in 5%. Ender et al. [2] evaluated augmented reality-enhanced 3D transesophageal echocardiography in 50 elective mitral valve repair patients using digital annuloplasty ring templates (28–36 mm); template-to-implanted ring correlation was 0.83 (post hoc validation correlation 0.94), with 60% exact matches and 38% within ±2 mm. In a porcine beating-heart model, Chu et al. [9] used a magnetically tracked AR environment to guide transapical NeoChord DS1000 navigation, achieving successful navigation in 12/12 attempts with AR versus 9/12 with TEE alone and reducing tool-tip trajectory error (5.2 ± 2.4 mm vs. 16.8 ± 10.9 mm), navigation time (16.7 ± 8.0 s vs. 92.0 ± 84.5 s), and path length (225.2 ± 120.3 mm vs. 1128.9 ± 931.1 mm). Physiological overlays were demonstrated by Szabó et al. [12] in five pigs using an infrared temperature mapping AR system during LAD occlusion, showing a distal temperature drop during ischemia (36.9 ± 0.60 °C baseline vs. 34.1 ± 1.66 °C ischemia) with return after reperfusion (37.4 ± 0.48 °C; *p* < 0.001). Postoperative translation was supported by the randomized trial of Ghlichi Moghaddam et al. [14] (*n* = 60), in which phase I CABG rehabilitation training delivered with AR significantly increased cardiovascular management self-efficacy compared with conventional rehabilitation (*p* < 0.001).

Within the primary evidence base, preclinical and technical investigations [8,9,10,11,13] frequently employed phantom, simulation, or animal models to evaluate navigation accuracy, registration fidelity, and the feasibility of ultrasound or temperature overlays. Across these studies, objective technical performance was most commonly reported as overlay/registration error, typically within the 2–5 mm range, alongside task-focused endpoints such as tool-target alignment and motion compensation during beating-heart conditions.

Clinical feasibility and early adoption studies [1,2,4,5,6,7,19] examined applications in minimally invasive CABG, mitral valve repair, and transcatheter procedures such as MitraClip implantation and balloon mitral commissurotomy. Across these reports, AR/MR was technically feasible and integrated into standard workflows, with quantitative or procedure-specific endpoints reported in selected contexts (e.g., annuloplasty ring sizing without prolonging bypass time, procedural feasibility and patency reporting in multicenter experience). Reported improvements in intraoperative orientation, decision-making, communication, and cognitive load were largely clinician- or user-reported rather than assessed using standardized objective instruments.

Overall, objective endpoints were most consistently available for technical accuracy and selected procedural metrics, whereas many human-factor benefits were described qualitatively. This heterogeneity in endpoints and measurement approaches limits cross-study comparability and reinforces the need for standardized outcome reporting in future evaluations.

Evidence from a randomized clinical trial [14] expands the scope of AR into the postoperative domain. In this study, an AR-based rehabilitation program for CABG patients led to significant improvements in cardiovascular self-efficacy compared with conventional rehabilitation, highlighting the potential of XR technologies to influence recovery as well as operative care. Complementary qualitative work [34] further suggests that XR-based rehabilitation is generally acceptable to patients, though barriers remain in older or frailer groups.

Integration with intraoperative imaging was a recurring theme across the primary literature. Ender et al. [2] showed that AR-enhanced TEE improved annuloplasty ring sizing during mitral valve repair without prolonging bypass time. Bainbridge et al. [11] demonstrated the feasibility of ultrasound overlays for off-pump intracardiac procedures, while Szabó et al. [12] visualized myocardial perfusion using real-time thermography. Kasprzak et al. [19] reported the first real-time holographic display of three-dimensional echocardiography during a structural intervention, providing a proof of principle for dynamic intraoperative holography.

Motion compensation strategies [13,17,32] addressed the challenge of cardiac and respiratory deformation. Virtual stabilization and robotic motion adaptation improved visualization for microscale surgical tasks, while learning-based deformable registration pipelines [27] suggest future pathways toward real-time compensation in clinical practice.

Beyond operative applications, additional work [15,16,21] evaluated AR/MR for preoperative planning and patient education, showing that interactive three-dimensional models improve patient comprehension of anatomy and procedural risk, reduce anxiety, and enhance shared decision-making. Systematic and narrative reviews [20,30,31] further contextualize these findings, emphasizing the translational potential of AR/MR across the surgical continuum.

Taken together, the evidence demonstrates that AR and MR are technically feasible in cardiac surgery and that objective technical performance has been reported most consistently in preclinical settings, while human-factor benefits related to orientation, cognitive load, and communication are largely described qualitatively. Nevertheless, most studies remain exploratory, limited by small cohorts, single-center designs, and short-term follow-up. Robust multicenter evaluations and standardized outcome reporting will be essential to establish clinical impact.

## 4. Discussion

### 4.1. Preoperative Planning and Patient-Specific Modelling

Mixed reality (MR) has been integrated into minimally invasive cardiac surgery (MICS) to project patient-specific 3D models, improving spatial orientation and operative confidence without prolonging operative time [5]. For complex valve and congenital scenarios, holographic reconstructions have been used to anticipate anatomical challenges and support multidisciplinary planning [3,6,7,15,16]. Collectively, these experiences underscore the value of immersive, patient-specific visualization to complement conventional imaging and operative checklists.

In addition to patient-specific cardiac reconstructions, preliminary assessment of thoracic morphology may provide complementary, low-burden anatomical context for preoperative planning. Available evidence suggests that individuals with a narrow anteroposterior thoracic diameter and concave chest wall conformation may exhibit more favorable outcomes and a lower risk of adverse cardiovascular events [37,38]. Because these morphometric features can be derived from routine preoperative imaging, systematic chest shape assessment could be integrated into preoperative workflows to support patient stratification and procedural planning, potentially identifying anatomical profiles that may benefit most from patient-specific modelling and immersive visualization. Further investigation is warranted to determine whether incorporating standardized chest morphology descriptors improves planning accuracy, approach selection, or downstream outcomes.

A parallel direction has been the incorporation of digital heart models derived from the Living Heart Project, which combine finite-element biomechanics with CT or MRI data to create deformable “digital twins.” Such models have been merged with XR displays to evaluate surgical approaches in silico before real interventions, allowing prediction of annular deformation after valve replacement or strain distribution after patch repair [17,18]. These computational surrogates extend the planning function of MR from static visual rehearsal to functional simulation, although they remain largely investigational.

Beyond surgeon preparation, preoperative visualization in XR may also enhance patient engagement. Recent studies indicate that interactive 3D models displayed through head-mounted devices or tablets improve patient comprehension of procedural risks and anatomical rationale compared with conventional two-dimensional imaging [17,19,20]. Patients exposed to these tools reported reduced anxiety and higher satisfaction with the informed consent process, suggesting an additional translational avenue where mixed reality could improve decision-making and trust in high-risk surgical pathways [17,19,20,21].

Finally, improvements in echocardiography-derived volumetric datasets and three-dimensional rotational angiography provide more readily available inputs for XR reconstruction. Integration of real-time 3D echocardiography into MR platforms has shown feasibility in structural interventions [17,22], suggesting that ultrasound-based models could provide dynamic, radiation-free planning tools, particularly for valve or congenital cases where CT is undesirable.

### 4.2. Intraoperative Navigation and Visualization

For beating-heart mitral interventions, preclinical platforms that register preoperative models to intraoperative imaging and tracked instruments have achieved overlay accuracies of approximately 5 mm, supporting intracardiac target localization [8,9]. While these technical accuracy results are encouraging, the available literature remains insufficient to directly link reported registration accuracy to operative efficiency, error rates, complications, or longer-term outcomes. Notably, several principles have translated into early clinical practice: holographic visualization of real-time 3D transesophageal echocardiography (TEE) during MitraClip implantation has been reported to improve operator orientation and team communication, whereas AR-enhanced TEE facilitated annuloplasty ring sizing without prolonging bypass or cross-clamp times [2,4,19]. Here, objective endpoints are procedure-specific when available (e.g., sizing accuracy or time neutrality), while communication/orientation benefits are primarily reported qualitatively.

Comparisons with conventional navigation emphasize potential benefits but also highlight challenges. In a pig model of beating-heart valve repair, AR-guided navigation produced more precise instrument placement and fewer target misses compared with fluoroscopy alone, although setup time and registration drift remained obstacles [9,23]. In early human feasibility studies, surgeons reported perceived reductions in cognitive load and clearer spatial relationships, particularly in minimally invasive approaches where tactile feedback and direct vision are limited [2,4,24].

Latency and registration stability remain crucial barriers. Overlay drift as small as a few millimeters can compromise safety when working near conduction tissue or coronary ostia. Prototype motion compensation frameworks that use surface tracking or predictive modeling have shown the ability to stabilize images in robotic beating-heart settings [13,25,26]. Recent deep learning-based deformable registration methods applied to cardiac MRI and echocardiography suggest that near real-time compensation of respiratory and cardiac motion is possible, although translation into a sterile intraoperative workflow is pending [17,27].

Multi-user and collaborative mixed-reality platforms also represent a promising evolution. Several groups have reported synchronized MR environments in which imagers, interventionalists, and surgeons can view and manipulate the same holographic model in real time, enhancing intra-team communication and procedural rehearsal [23,28,29]. Although these systems have not yet been validated in prospective cardiac surgical cohorts, they highlight how XR platforms may become integral to hybrid operating theatre workflows.

### 4.3. Physiological and Functional Guidance

AR has been extended beyond anatomy to functional monitoring. In a porcine model of LAD occlusion-reperfusion, Szabó and colleagues projected thermographic maps directly onto the myocardium, demonstrating an ischemic temperature drop with strong correlation to invasive thermometry and rapid normalization after reperfusion [12]. Such overlays provide immediate, radiation-free assessments of perfusion or graft patency during open procedures, with clear translational potential if incorporated into standard operative workflows. While the protective effect of preserving myocardium during ischemia is well established, the ability to monitor cardioplegia perfusion across the entire heart in real time—and adjust intraoperatively if needed—would markedly enhance procedural safety.

Other approaches have explored ultrasound-based AR overlays for closed, beating-heart navigation, supporting device placement without cardiopulmonary bypass [11,30]. The integration of Doppler flow data or near-infrared spectroscopy into augmented displays has also been piloted in animal studies, raising the possibility of real-time physiologic feedback to guide graft assessment or anastomotic revision. These methods remain preliminary but align with broader efforts in cardiac surgery to achieve intraoperative physiologic assurance without ionizing radiation or invasive probes [11,12,30].

Further clinical validation will be required to establish whether AR-derived functional information improves decision-making compared to current gold standards such as intraoperative TEE or flow wire measurements. Nevertheless, functional overlays represent one of the more distinctive frontiers of AR/MR, as they move the technology beyond purely spatial augmentation to dynamic physiologic integration.

### 4.4. Robotic Integration and Workflow Optimization

AR-guided port placement on phantoms optimized robotic instrument trajectories and reduced collisions, with registration errors on the order of millimeters [10]. Clinically, totally endoscopic CABG has been demonstrated in multicenter series, establishing feasibility for complex coronary procedures via telemanipulation [1]. In these contexts, overlays could be particularly useful for orientation within confined intrathoracic spaces where collisions between instruments are common.

Intraoperative imaging integration extends these capabilities. Ender et al. demonstrated that AR-enhanced TEE improved annuloplasty ring sizing during mitral valve repair by providing spatially registered overlays of annular anatomy, enabling more accurate device selection without prolonging bypass time [2]. More recently, reports of MR-based remote proctoring for complex valve interventions highlight how head-mounted displays can transmit synchronized volumetric views to remote experts, potentially reducing geographic barriers to program adoption [17,31].

Motion-stabilization frameworks that infer surface deformation to create a stabilized augmented view point toward future fiducial-less compensation during robotic beating-heart surgery [4,31,32]. At the same time, workflow studies emphasize the importance of stepwise training and team rehearsal when introducing AR/MR into robotic programs, where setup complexity and ergonomic demands can otherwise compromise efficiency [1,33].

### 4.5. Postoperative Rehabilitation

AR has also been evaluated in early postoperative care. In a randomized trial of phase-I rehabilitation after CABG, an AR-based program improved cardiovascular self-efficacy versus standard care, with no AR-related adverse events [14]. Qualitative work indicates that patients generally accept such interventions, although older or frailer cohorts may experience usability barriers related to headset weight, visual discomfort, or interface complexity [34].

These experiences suggest that AR-enabled rehabilitation could improve adherence and engagement in the vulnerable early recovery phase. Whether such programs translate into durable improvements in exercise tolerance, quality of life, or long-term readmission risk remains uncertain, but they illustrate that AR/MR applications in cardiac surgery extend across the perioperative continuum, from planning to recovery.

### 4.6. Cross-Cutting Limitations and Implementation Considerations

Across domains, most reports are feasibility-focused with small samples, single-center designs, and heterogeneous endpoints [3,4,5,6,7,14]. These features limit external validity and preclude meta-analysis. Key technical gaps include robust deformable registration for moving, deformable cardiac structures, latency constraints, and displays that are simultaneously ergonomic, sterile, and workflow-compatible [8,9,10,13,29]. Where we cite supporting XR literature beyond strictly operative cardiac series, it is to highlight enabling methods that address these cardiac-surgery-specific constraints and therefore materially affect translational feasibility. Accuracy thresholds for clinical safety remain procedure-dependent; errors tolerated for port placement may be unacceptable for leaflet repair or coronary anastomosis. In addition, outcomes are not assessed using standardized measures across studies; many perceived benefits (e.g., spatial orientation, cognitive load, workflow ease, and communication) are described qualitatively, limiting comparability across platforms and centers.

Notably, the current evidence base is dominated by small, feasibility-focused investigations, which increases vulnerability to selection bias and limits generalizability. Many studies are also susceptible to performance and learning-curve effects (e.g., outcomes influenced by operator familiarity with the platform), and to measurement bias when endpoints rely on unblinded assessments or non-standardized instruments. In addition, heterogeneity in study designs and endpoints complicates cross-study comparisons and may preferentially amplify positive impressions of utility. Publication bias is also plausible in an emerging technology field, as negative, null, or workflow-disrupting experiences may be less likely to be reported. Collectively, these considerations reinforce the need for multicenter evaluations with prespecified outcomes and transparent reporting standards.

Human factors are equally pressing. Head-mounted displays offer immersion but impose limitations in field of view, weight, sterilization, and prolonged wear, while projection-based or console-integrated displays may trade immersion for usability [3,4,7,19,23,35]. Cognitive load and surgeon fatigue require systematic evaluation, as prolonged reliance on overlays could introduce new forms of distraction or error. Team training and simulation will be necessary to ensure safe adoption, particularly in high-acuity settings.

Economic and regulatory considerations remain largely unaddressed. Few studies have evaluated the cost of AR/MR hardware, integration, and maintenance relative to potential efficiency gains or complication reductions [25,36]. Beyond acquisition, total cost of ownership may include software licensing, device tracking and calibration, sterile workflow adaptations, IT support, cybersecurity safeguards, imaging data handling, and team training time, all of which can meaningfully affect feasibility outside of early adopter centers. Without robust cost-effectiveness and budget-impact analyses grounded in objective endpoints (e.g., procedural time, error rates, complications, length of stay, or readmissions), adoption in resource-constrained settings will be difficult to justify.

Relatedly, regulatory and implementation pathways also require clearer definition. Regulatory frameworks for software-as-a-medical device are evolving, but most AR/MR platforms remain investigational, and clear guidance on validation standards, cybersecurity, and patient privacy for imaging-derived models is still needed. In parallel, real-world adoption barriers include interoperability with hospital imaging and operating-room systems, reliable low-latency performance, sterilization and ergonomics for prolonged use, onboarding of multidisciplinary teams, and governance for software updates and model versioning. Addressing these issues prospectively—alongside multicenter evidence generation—will be essential for translation from feasibility demonstrations to routine cardiac surgical care.

More broadly, AI-enabled wearable monitoring in cardiovascular medicine—such as blood pressure wearables that help uncover uncontrolled hypertension—illustrates how continuous sensing and analytics can close real-world care gaps [39].

## 5. Conclusions

AR and MR have progressed from experimental prototypes to early clinical applications in cardiac surgery. Preclinical work demonstrates millimetric overlays and motion stabilization in beating-heart settings, while early clinical reports suggest gains in visualization, orientation, and workflow integration without prolonging procedure times. Functional overlays and postoperative rehabilitation studies broaden the scope beyond anatomy. Yet translation into routine practice hinges on solving technical challenges of deformable registration and latency, addressing ergonomic and human-factor limitations, and generating robust multicenter evidence powered for efficiency, safety, and patient-centered outcomes. With continued advances in computational modeling, imaging integration, and human–machine interface design, XR platforms have the potential to become integral to the full perioperative continuum of cardiothoracic care.

## Figures and Tables

**Table 1:** Core studies evaluating augmented and mixed reality (AR/MR) in cardiac surgery across the perioperative continuum. Studies are grouped by evidence type (original clinical, preclinical/technical, and review evidence retained for contextual synthesis). For each publication, the table summarizes the setting/model, surgical indication, AR/MR modality, reported accuracy or outcome measures, and key findings. Abbreviations: AR, augmented reality; MR, mixed reality; VR, virtual reality; XR, extended reality; CABG, coronary artery bypass grafting; TECAB, totally endoscopic coronary artery bypass; TEE, transesophageal echocardiography; RCT, randomized controlled trial; N/A, not available.

Evidence Category	Ref	First Author, Year	Study Type	Setting/Model	Surgical Indication	AR/MR Modality	Accuracy/Outcome	Key Findings
(A) Original clinical evidence (human studies)								
	[1]	De Cannière, 2007	Multicenter clinical series	Human	Totally endoscopic CABG	Endoscopic/robotic	Procedural feasibility, patency	Demonstrated safety, feasibility in 148 pts
	[2]	Ender, 2008	Clinical feasibility	Human	Mitral valve repair	AR-enhanced TEE	Ring size accuracy	Improved sizing without time penalty
	[5]	Sacha, 2022	Clinical case	Human	MitraClip implantation	MR holography	Technical success	First-in-human demonstration of MR in MitraClip
	[6]	Winn, 2025	Comparative feasibility	Human	Minimally invasive cardiac surgery	MR holography	Task completion, workflow	MR reduced cognitive load, improved spatial awareness
	[7]	Nanchahal, 2022	Clinical feasibility	Human	Mitral valve surgery	VR/AR headset guidance	Integration success	Enabled enhanced anatomical visualization
	[12]	Szabó, 2013	Clinical feasibility	Human	Perfusion mapping	AR temperature overlay	Perfusion visualization	Enabled intraoperative myocardial perfusion assessment
	[14]	Ghlichi Moghaddam, 2023	RCT	Human	CABG rehabilitation	AR-based rehab training	Self-efficacy scores	Significant improvement vs. control
(B) Preclinical/technical evidence (phantom/animal/simulation; translational pilot where applicable)								
	[8]	Linte, 2007	Preclinical	Beating-heart phantom	Mitral valve surgery	VR/AR overlays	~5 mm registration error	Improved spatial orientation
	[9]	Chu, 2012	Preclinical + human pilot	Pig + clinical	Beating-heart mitral valve repair	AR navigation	Navigation accuracy	Improved repair navigation and tool positioning
	[10]	Bauernschmitt, 2006	Preclinical	Phantom	Robotic cardiac surgery	AR port placement	~2–5 mm accuracy	Optimized trajectories, avoided collisions
	[11]	Bainbridge, 2008	Preclinical	Animal	Off-pump intracardiac surgery	Ultra-sound-based AR	Tool-target alignment	Feasible guidance without open access
	[13]	Stoyanov, 2007	Preclinical	TECAB in vivo + simulation	Beating-heart surgery	Motion-stabilized AR	Motion compensation	Stabilized view improved precision tasks
(C) Review evidence								
	[3]	Rad, 2022	Narrative review with cases	Mixed	Cardiac surgery	VR/AR plat-forms	N/A	Summarized applications, technical aspects
	[4]	Sadeghi, 2020	Narrative review	N/A	Cardiothoracic surgery	XR	N/A	Highlighted current and future uses

## Data Availability

No new data were created or analyzed in this study.

## Supplementary Materials

The following supporting information can be downloaded at: https://www.mdpi.com/article/10.3390/jcm15031224/s1, Table S1: Summary of clinical, preclinical, and contextual studies evaluating augmented and mixed reality in cardiac surgery and related fields [1,2,3,4,5,6,7,8,9,10,11,12,13,14,15,16,17,18,19,20,21,22,23,24,25,26,27,28,29,30,31,32,33,34,35,36].

## Abbreviations

The following abbreviations are used in this manuscript:

AR	Augmented reality
AR/MR	Augmented reality/Mixed reality
CABG	Coronary artery bypass grafting
CT	Computed tomography
LAD	Left anterior descending (coronary artery)
MICS	Minimally invasive cardiac surgery
MR	Mixed reality
MRI	Magnetic resonance imaging
RCT	Randomized clinical trial
Suppl.	Supplementary
TECAB	Totally endoscopic coronary artery bypass
TEE	Transesophageal echocardiography
VR	Virtual reality
XR	Extended reality
