# Supplementary material for: Augmented and Mixed Reality in Cardiac Surgery: A Narrative Review
Source: J Clin Med. 2026 Feb 4;15(3):1224. doi: 10.3390/jcm15031224 (PMC12898313; doi:10.3390/jcm15031224)
Supplement: Supplementary file 1 [file jcm-15-01224-s001.zip › jcm-4125658-supplementary.pdf]

Supplementary Table 1. Summary of clinical, preclinical, and contextual studies evaluating augmented and mixed reality in cardiac surgery and related fields (Refs 1–36).

This table summarizes 36 publications relevant to augmented reality (AR) and mixed reality (MR) in cardiac surgery and perioperative care. References 1–14 represent the core body of original clinical and preclinical investigations directly applied to operative cardiac surgery. References 15–36 provide supporting and contextual evidence, including technical developments, imaging integration, patient education, systematic reviews, and translational applications from related disciplines. Information extracted includes study type, setting or model, surgical indication, AR/MR modality, accuracy or outcome measures, and principal findings. Abbreviations: CABG = coronary artery bypass grafting; TEE = transesophageal echocardiography; TECAB = totally endoscopic coronary artery bypass; MR = mixed reality; VR = virtual reality; XR = extended reality.

Ref	First author, year	Study type	Setting/model	Surgical indication	AR/MR modality	Accuracy / outcome measures	Key findings
1	De Cannière, 2007	Multicenter clinical series	Human	Totally endoscopic CABG	Endoscopic/robotic	Procedural feasibility, patency	Demonstrated safety, feasibility in 148 pts
2	Ender, 2008	Clinical feasibility	Human	Mitral valve repair	AR-enhanced TEE	Ring size accuracy	Improved sizing without time penalty
3	Rad, 2022	Narrative review with cases	Mixed	Cardiac surgery	VR/AR platforms	N/A	Summarized applications, technical aspects
4	Sadeghi, 2020	Narrative review	N/A	Cardiothoracic surgery	VR/AR/MR	N/A	Highlighted current and future uses
5	Sacha, 2022	Clinical case	Human	MitraClip implantation	MR holography	Technical success	First-in-human demonstration of MR in MitraClip
6	Winn, 2025	Comparative feasibility	Human	Minimally invasive cardiac surgery	MR holography	Task completion, workflow	MR reduced cognitive load, improved spatial awareness
7	Nanchahal, 2022	Clinical feasibility	Human	Mitral valve surgery	VR/AR headset guidance	Integration success	Enabled enhanced anatomical visualization
8	Linte, 2007	Preclinical	Beating-heart phantom	Mitral valve surgery	VR/AR overlays	~5 mm registration error	Improved spatial orientation
9	Chu, 2012	Preclinical + human pilot	Pig + clinical	Beating-heart mitral valve repair	AR navigation	Navigation accuracy	Improved repair navigation and tool positioning
10	Bauernschmitt, 2006	Preclinical	Phantom	Robotic cardiac surgery	AR port placement	~2–5 mm accuracy	Optimized trajectories, avoided collisions
11	Bainbridge, 2008	Preclinical	Animal	Off-pump intracardiac surgery	Ultrasound-based AR	Tool–target alignment	Feasible guidance without open access
12	Szabó, 2013	Clinical feasibility	Human	Perfusion mapping	AR temperature overlay	Perfusion visualization	Enabled intraoperative myocardial perfusion assessment
13	Stoyanov, 2007	Preclinical	TECAB in vivo + simulation	Beating-heart surgery	Motion-stabilized AR	Motion compensation	Stabilized view improved precision tasks
14	Ghlichi Moghaddam, 2023	RCT	Human	CABG rehabilitation	AR-based rehab training	Self-efficacy scores	Significant improvement vs control
15	Grab, 2023	Observational/feasibility	Human	Pre-op patient education	3D printing + VR (XR)	Patient comprehension/anxiety (reported)	Describes improved patient understanding and counseling utility
16	Minga, 2024	Narrative review	Mixed	Cardiovascular practice	VR/AR + 3D models	N/A	Overview of contemporary uses across cardiology
17	Peoples, 2019	Technical / methods	Software/phantom	Beating-heart navigation	Deformable multimodal registration	Method validation (registration performance)	Demonstrated feasibility of deformable US–CT registration for navigation

Ref	First author, year	Study type	Setting/model	Surgical indication	AR/MR modality	Accuracy / outcome measures	Key findings
18	Segars, 2019	Technical	Simulation (XCAT + LHM)	Cardiac imaging research	Computational "Living Heart" model	Creation of 4D deformable phantom	Integrated LHM into XCAT enabling realistic motion/deformation studies
19	Kasprzak, 2019	First-in-man case	Human	Balloon mitral commissurotomy	Real-time MR display of 3D TEE	Feasibility	Real-time holographic 3D echo display feasible during intervention
20	Magalhães, 2024	Systematic review	Mixed	Operating room (surgery, multi-specialty)	Mixed reality (MR)	N/A	Synthesizes MR in the OR, workflow/ergonomics themes
21	Evans, 2025	Systematic review	Mixed	Consent/patient education (surgical)	AR (XR)	Comprehension/consent process outcomes (reported)	AR tools can enhance consent/education; heterogeneous evidence
22	Arujuna, 2014	Preclinical + clinical feasibility	Animal + human	Image-guided cardiac interventions	Real-time 3D echo–fluoroscopy fusion	Preclinical validation; clinical feasibility	Demonstrated integrated 3D TEE–fluoro guidance platform
23	Finos, 2024	First clinical use report	Human	Interventional radiology (non-cardiac)	MR navigation platform (XR90)	Procedural feasibility	Demonstrated initial clinical deployment in IR; translational relevance
24	Annabestani, 2024	Review	Mixed	Interventional cardiology	Mixed/augmented reality	N/A	Contemporary review of AR/MR in cath-lab practice
25	Sadeghi, 2024	Perspective/technical note	Human (thoracic)	Robotic lung surgery	AI-assisted AR	N/A	Describes AR integration concepts transferable to cardiac robotics
26	Roshanfar, 2025	Review	Mixed	Cardiac interventions (robotics)	Advanced robotics (incl. XR context)	N/A	Reviews next-gen robotics relevant to AR-guided interventions
27	Zakeri, 2023	Technical (imaging)	MRI sequences	Cardiac cine generation	Learning-based deformable registration	Algorithmic performance (imaging)	Presents DL deformable registration framework (supports motion compensation)
28	Anabtawi, 2024	Technical/feasibility	Human (open surgery)	Telementoring (general surgery)	Holographic telementoring (instrument overlay)	Qualitative/operational metrics (reported)	Feasible real-time depiction of instrument motion for guidance
29	Beitnes, 2015	Clinical feasibility	Human echo datasets	Mitral valve analysis	3D ultrasound to holographic screen	Feasibility/usability	Demonstrated holographic rendering for MV analysis
30	Tsai, 2023	Review	Mixed	Cardiovascular medicine	Extended realities (XR)	N/A	Broad review of XR across CV medicine
31	Jung, 2022	Review	Mixed	Cardiovascular care	VR/AR	N/A	Synthesis of VR/AR applications and challenges in CV care
32	Yuen, 2009	Preclinical/engineering	Robotic platform	Beating-heart intracardiac surgery	Robotic motion compensation	Compensation accuracy/latency (reported)	Demonstrated robotic motion compensation on beating tissue
33	Riad, 2025	Review	Mixed	Robotic surgery (general)	—	N/A	Holistic operational/implementation review (adoption, team dynamics)
34	Wu, 2025	Qualitative study	Human (post-op)	Early rehab after cardiac surgery	Commercial VR games	Acceptability/usability themes	Patients generally accept VR; usability barriers noted
35	Shuhaiber, 2004	Early commentary/review	Mixed	Surgery (general)	Augmented reality	N/A	Early perspective on AR in surgery; foundational context
36	Alam, 2025	Review (industry)	Non-medical (Industry 4.0)	Maintenance operations	XR technologies	N/A	Trends/challenges in XR outside medicine; transferable ergonomic insights
